# Cloning, Expression, Characterization, and Antioxidant Protection of Glutaredoxin3 From Psychrophilic Bacterium *Psychrobacter* sp. ANT206

**DOI:** 10.3389/fmicb.2021.633362

**Published:** 2021-04-08

**Authors:** Yatong Wang, Yanhua Hou, Quanfu Wang

**Affiliations:** ^1^School of Environment, Harbin Institute of Technology, Harbin, China; ^2^School of Marine Science and Technology, Harbin Institute of Technology, Weihai, China

**Keywords:** antarctic, cold-adapted, expression, homology modeling, site-directed mutagenesis, antioxidant protection

## Abstract

Glutaredoxins (Grxs) are proteins that catalyze the glutathione (GSH)-dependent reduction of protein disulfides. In this study, a Grx-related gene (264 bp), encoding a Ps-Grx3, was cloned from *Psychrobacter* sp. ANT206. Sequence analysis indicated the presence of the active site motif CPYC in this protein. Homology modeling showed that Ps-Grx3 had fewer hydrogen bonds and salt bridges, as well as a lower Arg/(Arg + Lys) ratio than its mesophilic homologs, indicative of an improved catalytic ability at low temperatures. Site-directed mutagenesis demonstrated that the Cys13, Pro14, and Cys16 sites were essential for the catalytic activity of Ps-Grx3, while circular dichroism (CD) spectroscopy confirmed that point mutations in these amino acid residues led to the loss or reduction of enzyme activity. Furthermore, analysis of the biochemical properties of Ps-Grx3 showed that the optimum temperature of this enzyme was 25 °C. Importantly, Ps-Grx3 was more sensitive to tBHP and CHP than to H_2_O_2_, and retained approximately 40% activity even when the H_2_O_2_ concentration was increased to 1 mm Regarding substrate specificity, Ps-Grx3 had a higher affinity for HED, L-cystine, and DHA than for S-sulfocysteine and BSA. We also investigated the DNA-protective ability of Ps-Grx3 using the pUC19 plasmid, and found that Ps-Grx3 could protect supercoiled DNA from oxidation-induced damage at 15°C for 1.5 h. This study provides new insights into the structure and catalytic activity of a cold-adapted Grx3.

## Introduction

Grxs form part of the glutaredoxin system, which is a thiol-dependent reductase system involved in the regulation of the thiol redox state (Ouyang et al., 2018). Several Grxs are known to play crucial roles in iron-sulfur cluster biogenesis, a fundamental process given the importance of iron-sulfur proteins in cellular physiology (Rouhier et al., 2010). Grxs are proposed to be involved in a variety of cellular processes, including the synthesis of deoxyribonucleotides, the generation of reduced sulfur, signal transduction, and the defenses against oxidative stress (Fernandes and Holmgren, 2004). In general, the functions of Grxs are closely associated with the GSH system, Grxs catalyze thioldisulfide exchange reactions with GSH, generating oxidized glutathione (GSSG), which is then reduced by glutathione reductase (GR). In addition, they are highly efficient at catalyzing the reversible S-glutathionylation of specific proteins (Ebersoll et al., 2018). Grxs can be broadly separated into two major subfamilies, termed class I and II Grxs, as well as several less prevalent and smaller subfamilies. Class I Grxs exhibit oxidoreductase activities, and typically contain two active-site cysteine residues, such as that found in the CPYC motif (Deponte, 2013). Class II Grxs are involved in the regulation of iron metabolism and in the maturation of iron-sulfur proteins (Couturier et al., 2013; Haunhorst et al., 2013) and usually contain one active-site cysteine residue, such as that present in the CGFS motif (Couturier et al., 2009). The terms “dithiol” and “monothiol” Grx have sometimes been used as alternative names for class I and class II Grxs, respectively (Zimmermann et al., 2020). Eukaryotic Grx3 isoforms are primarily monothiolic, and are reported to exert redundant and additive functions in cell growth and protection against oxidative stress (Ye et al., 2019). In contrast, in prokaryotes, Grx3 isoforms are mainly dithiolic, and mediate the activity of complex redox pathways in bacterial cells (Shekhter et al., 2010). In addition, the structure and several functions of Grx3 have been revealed through mutational analysis (Nordstrand et al., 1999b; Shi et al., 1999; Elgan et al., 2010).

The environmental conditions found in Antarctica, which include cold, low light, high salinity, and strong radiation, are conducive to the generation of reactive oxygen species (ROS) in living organisms. This environment can damage nucleic acids and biofilms, thereby seriously affecting the normal transcription and translation processes and leading to energy metabolism disorders in cells (Sies, 2017). To survive in a harsh environment, microorganisms have evolved complex antioxidant systems, such as the glutaredoxin system, that are vital for the maintenance of oxidative homeostasis (Chattopadhyay et al., 2011). There is some evidence to support that Grxs exert a protective role under oxidative conditions. For instance, they have been shown to play distinct roles in the sensitivity of cells to oxidative stressors (Allen and Mieyal, 2012; Mailloux and Treberg, 2016). Although the structure and function of most Grxs have been characterized, few studies have reported on cold-adapted Grx3s. Here, we purified and expressed a novel Grx3 from *Psychrobacter* sp. ANT206, an Antarctic sea-ice bacterium, and further revealed its biochemical and antioxidant properties. Our findings indicated that Ps-Grx3 is a new type of cold-adapted enzyme with essential roles in combating oxidative stress.

## Materials and Methods

### Strains and Material

*Psychrobacter* sp. ANT206 (*P.sp.* ANT206) was collected from the Antarctic sea ice (GenBank accession number: MK968312) and kept frozen at −80°C until use. The *Escherichia. coli* (*E.coli*) BL21 strain and the pET-28a(+) plasmid were kept in our laboratory. The PCR reagents were obtained from TaKaRa Bio (Dalian, China). 2-Hydroxyethyl disulfide (HED), GR, nicotinamide adenine dinucleotide phosphate (NADPH), GSH, diethyldithiocarbamate (DDC), sodium azide (NaN_3_), dithiothreitol (DTT), hydrogen peroxide (H_2_O_2_), tert-butyl hydroperoxide (tBHP) and cumene hydroperoxide (CHP) were purchased from Sigma-Aldrich (Shanghai, China). All the other reagents were acquired from Sinopharm (Beijing, China), and were of analytical grade or higher.

### Identification of the Ps-grx3 Gene

Based on the sequence and annotation of the genome of *Psychrobacter.* sp. ANT206, the *grx3* gene was amplified by PCR using the forward primers: 5′-GACGGATCCATGACTGTATCTGTTA-3′, which contained a unique *Bam*HI restriction site; and the reverse primer 5′-CATAAGCTTTGCTCGATAATCGCCC-3′ containing a unique *Hin*dIII restriction site. The PCR mix contained 0.2 μL (250 U) of Taq DNA polymerase, 1 μL of dNTPs (2.5 mM), 0.5 μM each primer, 25 ng of genomic DNA, 2 μL of 10 × PCR buffer (with Mg^2+^), and PCR-grade water to a final volume of 20 μL. PCR was performed with an initial denaturation at 94°C for 5 min, followed by 30 cycles at 94°C for 1 min, 57.1°C for 1 min and 72°C for 90 s, with a final extension at 72°C for 10 min. The phylogenetic tree was established using Mega 5.2 and sequences were obtained from NCBI. Multiple sequence alignments were performed using the BioEdit and ESPript programs.

### Homology Modeling and Binding Energy Interactions of Ps-Grx3

The 3D structure of Ps-Grx3 was obtained based on its amino acid sequence^1^ (Kelley et al., 2015), and the quality of the model was evaluated by Ramachandran plot generated by PROCHECK^2^. The software PyMOL was employed for homology modelings and to analyze the 3D model of the target proteins. Structural adaptation features were predicted based on previously described methods (Tina et al., 2007). The GSH 3D structure was obtained from the Zinc database and Open Babel was used to change the sdf file to a pdb file format. GSH was chosen for studying molecular docking (protein-ligand) interactions. Interactions between Ps-Grx3 and its ligand and between EcGrx3 (PDB ID: 1FOV) and its ligand were analyzed by AutoDock Vina (Trott and Olson, 2010). AutoDock Vina was run several times to obtain various docking configurations. The parameters were used for Ps-Grx3 and EcGrx3 were as follows: center *x* = 7.280; *y* = 0.479, *z* = 15.903, size *x* = 1.147; *y* = −0.037; and *z* = 0.525.

### Expression and Purification of the Recombinant Ps-Grx3

Plasmids containing recombinant Ps-Grx3 and its mutant forms were expressed in *E. coli* and purified as previously described (Wang et al., 2019). The recombinant plasmids were transformed into competent *E. coli* BL21 for Ps-Grx3 expression. *E. coli* BL21 harboring the recombinant plasmids were inoculated into LB liquid medium supplemented with kanamycin (100 mg/L) and cultured at 37°C to an OD_600_ of 0.5-0.6. Then, IPTG was added to a final concentration of 1.0 mM, and the mixture was incubated for 12 h at 25°C. Next, the induced cells were treated with 8 M urea overnight to generate inclusion bodies. Finally, the protein solutions were centrifuged, and the supernatants, which contained crude Ps-Grx3 extract or those of its mutant forms, were collected. The supernatants were then diluted 30 times in PBS (pH 8.0) and kept at 25°C for 2 h for protein refolding. Ps-Grx3 and its mutant forms were purified by Ni-NTA affinity chromatography. The samples were eluted with 50 mM imidazole buffer (20 mM Tris–HCl, 500 mM NaCl, pH 8.0) at a flow rate of 1 mL/min and stored at −20°C. The purification fold was obtained by the ratio of the specific activity of purified Ps-Grx3 to that of the crude enzyme. The molecular mass of Ps-Grx3 was estimated by SDS-PAGE (12.5% polyacrylamide gels).

### Grx Activity Assay and Protein Determination

The standard HED assay was used to measure enzyme activity as previously described (Zhang et al., 2017) with some modifications. The reaction mixture consisted of 100 mM Tris–HCl (pH 8.0), 0.6 μg/mL GR, 0.5 mM HED, 0.2 mM NADPH, 1.0 mM GSH, and 0.12 μg/mL purified protein in a total volume of 200 μL. The changes in absorbance were determined at 340 nm and 25°C. One unit of Ps-Grx3 activity was defined as the amount of Ps-Grx3 needed to oxidize 1 μmol of NADPH at 25°C every minute. Protein concentrations were determined by the Bradford method (Bradford, 1976).

### Site-Directed Mutagenesis and Circular Dichroism Spectroscopy

The Cys13, Pro14, Tyr15, Cys16, Arg43, Thr55, Val56, and Pro57 residues in the active sites and binding sites were mutated to evaluate the effects of these residues on enzyme activity. To generate mutant forms of Ps-Grx3, the pET-28a(+) vector was used as a template for the site-directed mutagenesis of the Cys13, Pro14, Tyr15, Cys16, Arg43, Thr55, Val56, and Pro57 residues to alanine using the QuikChange Site-directed Mutagenesis Kit (Stratagene) following the manufacturer’s instructions. The primers designed for site-directed mutagenesis are shown in Table 1. The expression and purification of the mutant proteins were performed as described for wild-type Ps-Grx3.

**TABLE 1:** Primer for Ps-Grx3 mutant forms.

**Name**	**Primer sequences**	**Derection**
C13A F	5′-TCTGTTAAAGTTTATACTACTCCTATCGCCCCATATTGCTCAAATGCTAAG-3′	Forward
C13A P	5′-CTTAGCATTTGAGCAATATGGGGCGATAGGAGTAGTATAAACTTTAACAGA-3′	Reverse
P14 A F	5′-GTTTATACTACTCCTATCTGCGCATATTGCTCAAATGCTAAGC-3′	Forward
P14 A R	5′-GCTTAGCATTTGAGCAATATGCGCAGATAGGAGTAGTATAAAC-3′	Reverse
Y15A F	5′-TTATACTACTCCTATCTGCCCAGCTTGCTCAAATGCTAAGCAACTG-3′	Forward
Y15A R	5′-CAGTTGCTTAGCATTTGAGCAAGCTGGGCAGATAGGAGTAGTATAA-3′	Reverse
C16A F	5′-TACTACTCCTATCTGCCCATATGCCTCAAATGCTAAGCAACTG-3′	Forward
C16A R	5′-CAGTTGCTTAGCATTTGAGGCATATGGGCAGATAGGAGTAGTA-3′	Reverse
R43A F	5′-CATTAAAGCACGGGCATCATCGCTGCTCATATCGTGCAT-3′	Forward
R43A R	5′-ATGCACGATATGAGCAGCGATGATGCCCGTGCTTTAATG-3′	Reverse
T55A F	5′-CTTTAATGCAAAAGACTAATAACTATCGTGCCGTGCCACAAATCT-3′	Forward
T55A R	5′-AGATTTGTGGCACGGCACGATAGTTATTAGTCTTTTGCATTAAAG-3′	Reverse
V56A F	5′-CTAATAACTATCGTACCGCGCCACAAATCTTCGTCGG-3′	Forward
V56A R	5′-CCGACGAAGATTTGTGGCGCGGTACGATAGTTATTAG-3′	Reverse
P57A F	5′-ACCGACGAAGATTTGTGCCACGGTACGATAGTTAT-3′	Forward
P57A R	5′-ATAACTATCGTACCGTGGCACAAATCTTCGTCGGT-3′	Reverse

For all Circular Dichroism (CD) measurements, five repeated scans were carried out to establish a baseline. Far-UV CD measurements were performed in a 0.1-cm quartz cuvette (Hellma, Germany) between 260 and 180 nm for 12 s every 1.0 nm using a bandwidth of 1.5 nm in a Jasco J-107 spectropolarimeter (Jasco, Japan). Ps-Grx3 and its mutant forms were separately diluted in a solution of 100 mM Tris–HCl (pH 8.0). Changes in secondary structure were presented as molar ellipticity [θ], according to the following equation: to the following equation:

$${\left[\theta \right]}_{\lambda }=\frac{\theta }{n*l*10*c}$$

in which θ is the ellipticity measured at a given wavelength *λ* (deg), *n* is the number of amino acids, *l* is the cell path length (cm), and *c* is the protein concentration (mol/L).

### The Temperature Dependence of Ps-Grx3 Activity and It’s Stability Profile

The optimum temperature for Ps-Grx3 activity was evaluated at different temperatures (0–60°**C**). To understand how temperatures influences Ps-Grx3 stability, the enzyme was incubated in a 100 mM Tris–HCl buffer (pH 8.0) solution for 1 h at 30, 40, and 50°C. Residual activity was measured as mentioned above.

### The Effects of Different Reagents and Chemicals

The purified enzyme was incubated in 100 mM Tris–HCl buffer for 30 min to detect the effects of different ions and chemicals on its activity. After a 30-min pre-incubation, residual activity was compared to that of the enzyme incubated without any reagent. To measure the effects of chemicals such as DDC, NaN_3_, DTT, H_2_O_2_, tBHP, and CHP, purified Ps-Grx3 was preincubated with 0.5 mM of each chemical for 30 min. Enzyme activity was measured as described above. Based on the results, we further explored the effect of different concentrations of H_2_O_2_ (0–1.0 mM) on the activity of purified Ps-Grx3.

### The Kinetics Parameters of Ps-Grx3

The reaction mixture contained 0.6 μg/mL GR, 0.2 mM NADPH, and 1.0 mM GSH in 100 mM Tris–HCl buffer (pH 8.0), and various amounts of substrates (HED, bovine serum albumin BSA, dehydroascorbate DHA, S-sulfocysteine or L-cystine) at different concentrations (0.5 mM to 2.0 mM) in a total volume of 200 μL. Changes in absorbance at 340 nm were measured at 25 °C. The same amount of purified protein was used for all the assays. The *K*_*m*_, *V*_*m*_, *k*_*cat*_, and *k*_*cat*_/*K*_*m*_ values were obtained at 25°C based on previously described methods (Lonhienne et al., 2000). The above experiments were performed three times.

### Metal-Catalyzed Oxidation Assay of Ps-Grx3

To evaluate the ability of Ps-Grx3 to protect supercoiled DNA, an Metal-Catalyzed Oxidation (MCO) DNA cleavage protection assay was performed as previously described (Revathy et al., 2012). The reaction mixture contained 30 μM FeCl_3_, 3 mM DTT, and 6.25–50 μg/mL purified protein/BSA in a total volume of 100 μL, and the assay was performed at 25°C for 2.5 h. Next, 1 μg of pUC19 supercoiled DNA was added to the mixture, and incubation was continued for 1.5 or 2.5 h at 15°C and 25°C. Finally, the reaction mixture was purified and the sample was evaluated on a 1% agarose gel. The same reaction conditions were used throughout the assay.

## Results and Discussion

### Identification of the Ps-grx3 Gene

**FIGURE 1:**
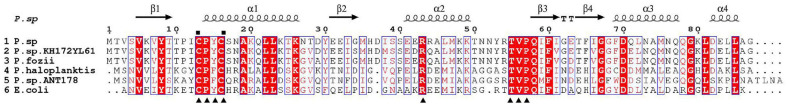
Alignment of the Ps-Grx3 sequence with those of other Grxs. *P.sp*, *Psychrobacter* sp. ANT206 Grx3 (GI: MW245063); *P.sp.*KH172YL61, *Psychrobacter sp.* KH172YL61 Grx3 (GI: BBI67010); *P.fozii*, *Psychrobacter fozii* Grx3 (GI: PYE39608); *P. haloplanktis*, *Pseudoalteromonas haloplanktis* Grx3 (GI: OLF76599); *P.sp*.ANT178, *Pseudoalteromonas* sp. ANT 178 Grx3 (GI: KF361316); and *E.coli*, *Escherichia coli* K-12 Grx3 (PDB ID: 1FOV). The conserved catalytic sites are indicated with squares and the residues involved in GSH binding sites are indicated with triangles.

The phylogenetic tree showed that Grx from *Psychrobacter* sp. ANT206 was indeed a class I Grx3 (Supplementary Figure 1). The open reading frame (ORF) of Ps-Grx3 (GenBank accession number: MW245063) contained 264 bp and encoded an 87-residue protein, while Grx from *Taenia solium* consisted of 315 bp and coded for a 105-amino acid protein (Nava et al., 2019). Sequence alignment (Figure 1) indicated that Cys13 and Cys16 were the catalytic sites and that Ps-Grx3 contained a redox-active site (CPYC motif), indicating that Ps-Grx3 is a dithiol Grx. The amino acid sequence of Grx3 from *E. coli* K-12 (EcGrx3, PDB ID: 1FOV) consists of 82 residues and contains a redox-active motif, Cys-Pro-Tyr-Cys, typical of the glutaredoxin family (Aslund et al., 1996). Additionally, Ps-Grx3 exhibited, respectively, 94.25 and 90.80% sequence similarity with *Psychrobacter sp.* KH172YL61 Grx3 and *P.fozii* Grx3.

### Homology Modeling and 3D Structure Analysis

**FIGURE 2:**
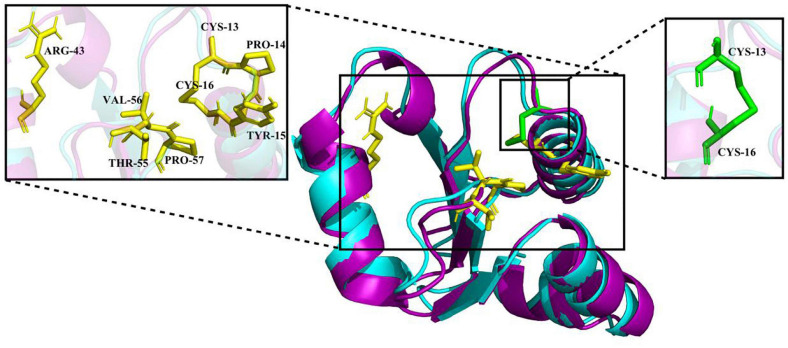
A 3D structure model of Ps-Grx (purple) superimposed on EcGrx3 (cyan, PDB ID: 1FOV). The GSH binding motifs and active sites are indicated as stick models colored in yellow and green, respectively.

The crystal structure of EcGrx3 from *E. coli* K-12 (PDB ID: 1FOV) was used as a template to elucidate the structure of Ps-Grx3 (Figure 2). The 3D model was verified by Ramachandran plot analysis, and the parameters indicated that the structural model of Ps-Grx3 was valid. The 3D structures of Ps-Grx3 and EcGrx3 were compared and showed good overlap (Figure 2).

To explore the cold-adaptive characteristics of Ps-Grx3, we compared it with its homolog EcGrx3. Compared with EcGrx3 (61), Ps-Grx3 has fewer hydrophobic interactions (50) (Table 2), which are known to be related to rigidity. Fewer hydrophobic bonds might lessen the rigidity of Ps-Grx3, thereby reducing its structural stability and greatly increasing its flexibility (Li et al., 2018). Further, Ps-Grx3 and EcGrx3 formed 70 and 1 versus 84 and 3 hydrogen bonds and salt bridges, respectively. The stability of cold-adaptive proteins is related to the number of both hydrogen bonds and salt bridges, and cold-adaptive enzymes usually have fewer of both than their mesophilic counterparts (Zheng et al., 2016), which was also consistent with the results of this study. Furthermore, as reported for other cold-adapted enzymes (Fu et al., 2013; Wang et al., 2020), the Arg/(Arg + Lys) ratio of Ps-Grx3 (0.33) was lower than that of EcGrx3 (0.45), which might be due to the increased conformational flexibility that would be expected to improve its catalytic ability under low temperatures (Feller and Gerday, 2003).

**TABLE 2:** Comparison of structural adaptation features between Ps-Grx3 and EcGrx3.

	**Ps-Grx3**	**EcGrx3**
Hydrogen bonds	70	84
Salt bridges	1	3
Cation-Pi interactions	0	1
Aromatic interactions	4	4
Hydrophobic interactions	50	61
Arg/(Arg+Lys)	0.33	0.45
R (Arg)	3 (3.4%)	5 (6.0 %)
P (Pro)	3 (3.4%)	4 (4.8 %)
G (Gly)	7 (8.0%)	7 (8.4 %)

Molecular docking analysis is commonly used to predict ligand-protein binding modes (Hossain et al., 2016). Here, we used AutoDock Vina to compare the affinity of Ps-Grx3 and EcGrx3 (PDB ID: 1FOV) for GSH and the interaction between Ps-Grx3 or EcGrx3 and GSH. As shown in Table 3, the highest and lowest binding energy values for the interaction between GSH and Ps-Grx3 or EcGrx3 were determined to be −4.9 and −4.4 kcal/mol, respectively, for Ps-Grx3; and −5.0 and −4.4 kcal/mol, respectively, for EcGrx3. This indicated that Ps-Grx3 and EcGrx3 had similar affinities for GSH.

**TABLE 3:** Binding energy values for the interaction between Ps-Grx3 or EcGrx3 and GSH.

**Pose mode**	**Docking scores based on kcal/mol *via* the GSH interactions**	
	
	**Ps-Grx3**	**EcGrx3**
1	−4.9	−5.0
2	−4.7	−5.0
3	−4.7	−4.9
4	−4.7	−4.9
5	−4.7	−4.9
6	−4.6	−4.6
7	−4.6	−4.6
8	−4.4	−4.5
9	−4.4	−4.4

### Expression, Purification, and Enzyme Assays of Ps-Grx3

**FIGURE 3:**
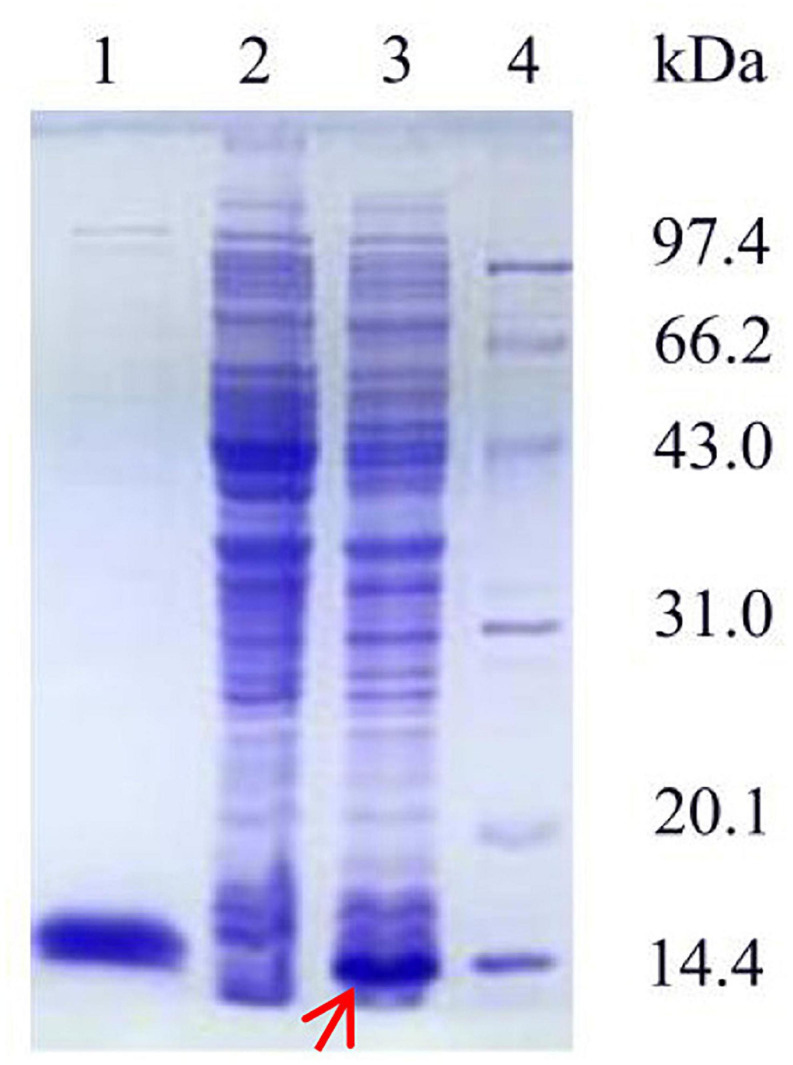
SDS-PAGE analysis of the expression and purification of recombinant Ps-Grx3. Lane 1, purified Ps-Grx3 after Ni-NTA affinity chromatography; lane 2, crude extract from the *E. coli* BL21/pET-28a(+); lane 3, crude extract from the *E. coli* BL21/pET-28a(+)-Ps-Grx3; lane 4, protein molecular weight marker.

The recombinant plasmid pET-28a(+) was transfected into *E. coli* BL21. As shown in Figure 3, the SDS-PAGE profile of the purified Ps-Grx3 enzyme demonstrated a single major band (∼13.8 kDa), The purification factor for Ps-Grx3 was 6.14-fold and the recovery yield was 67.5% (Table 4). Additionally, the specific activity of Ps-Grx3 was 217.88 U/mg, while that of *E. coli* K-12 Grx3 was 410 U/mg (Nordstrand et al., 1999a).

**TABLE 4:** The purification of Ps-Grx3.

**Step**	**Total protein (mg)**	**Total activity (U)**	**Specific activity (U/mg)**	**Yield in activity (%)**	**Purification (fold)**
Crude extraction	116.54	4134.87	35.48	100	1
Ni-NTA column purification	12.81	2791.04	217.88	67.5	6.14

### Site-Directed Mutagenesis and CD Spectroscopy

**FIGURE 4:**
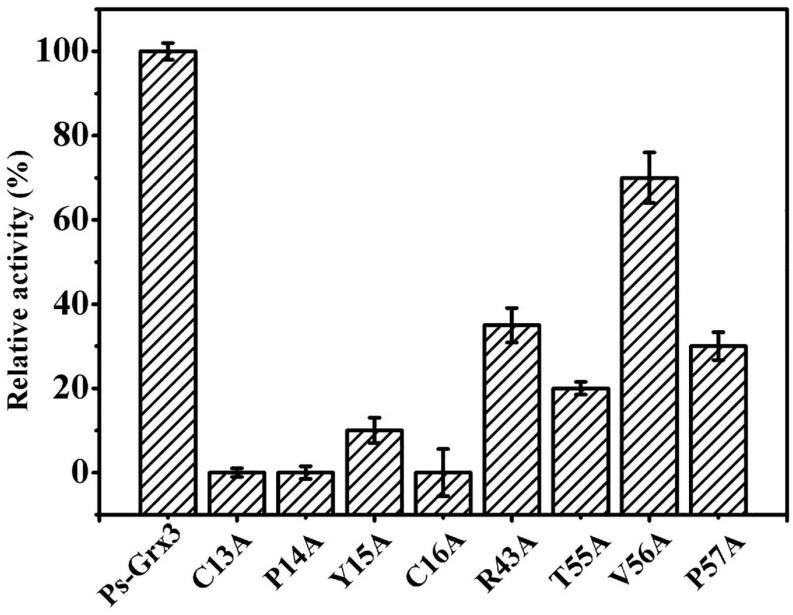
Measurement of the relative activity of Ps-Grx3 mutant forms generated by site-directed mutagenesis. The activity of the enzyme without mutation was defined as 100%. The experiments were carried out three times.

The activity of Ps-Grx3 and that of its mutant forms was examined using HED as a substrate. Among all the mutants, the R43A, T55A, and P57A forms exhibited the largest reduction in enzyme activity (65, 80, and 70%, respectively), indicating that these residues might be involved in the catalytic activity of the enzyme (Figure 4). Importantly, the C13A, P14A, and C16A forms lost all enzyme activity, which demonstrated that these residues are likely to be crucial for catalysis. In *E. coli*, the C14S, C14A, and H15V class I Grx3 mutants exhibit reduced activity, while the Cys-14 to Ala mutation results in a complete loss of activity (Nordstrand et al., 1999b). Moreover, the *E. coli* Grx3 C15S mutant retains near-total activity, whereas the Grx3 C12S mutant shows no activity (Shi et al., 1999), Additionally, the Grx3 C65Y mutation does not affect the *in vivo* activity of the enzyme (Elgan et al., 2010). Importantly, the C13, P14, and C16 residues are conserved in Ps-Grx3, and Grx from *Taenia solium* has a conserved dithiol C34PYC37 active site (Nava et al., 2019). Furthermore, two catalytic sites—Cys25 and Cys28—are also present in *Chlorella sorokiniana* T-89 Grx (Chuang et al., 2014).

**FIGURE 5:**
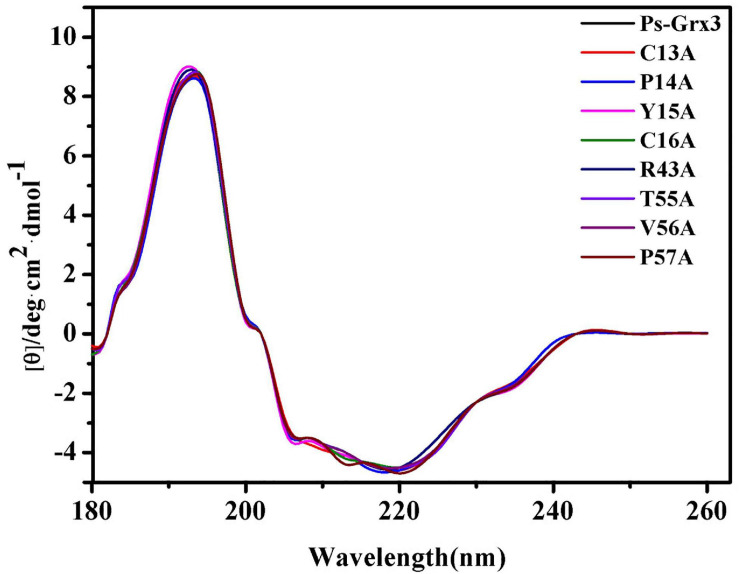
Far-ultraviolet (UV) circular dichroism (CD) spectra from 180 to 260 nm of Ps-Grx3 and its mutant forms.

CD-spectroscopy was used to confirm that the secondary structure of the mutant forms of Ps-Grx3 had not changed and to ensure that the loss or weakening of the activity of the mutant enzyme was indeed due to point mutations in key amino acid residues rather than changes in secondary structure. The mutations used throughout this study do not appear to have affected the conformation of the respective proteins, as judged by CD spectrometry results (Figure 5), indicating that C13, P14, and C16 of Ps-Grx3 were active sites and played an important role in its activity.

### The Temperature Dependence of the Activity of Ps-Grx3 and Its Stability

**FIGURE 6:**
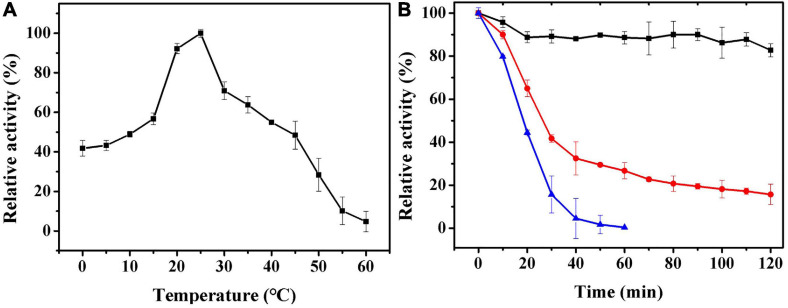
**(A)** The optimal temperature of Ps-Grx3 was determined by measuring its activity at temperatures from 0 to 60°C. **(B)** The effect of temperatures on the stability of the purified Ps-Grx3. The enzyme was incubated at 30 (■), 40 (⚫), and 50 °C (▲) for 120 min. Its activity relative to time zero was set as 100%. The above experiments were carried out three times.

The effect of temperature on Ps-Grx3 enzyme activity is shown in Figure 6A. Analysis of the temperature dependence of Ps-Grx3 showed that the enzyme exhibited optimal activity against HED at 25°C. The optimal temperature for *Chlorella sorokiniana* Grx is 50°C (Chuang et al., 2014). In the thermal inactivation assay, Ps-Grx3 retained almost 40% residual activity at incubations between 0 and 10°C, was highly stable from 0 to 30°C, and retained a greater than 90% residual activity after 90 min at 30°C (Figure 6B). After 30 min at 40°C, Ps-Grx3 lost approximately 60% of its activity, irreversible denaturation occurred after 30 min at 50°C, and its half-life was 18 min at 50°C. In comparison, Grx3 from *Pseudoalteromonas* sp. ANT178 lost almost all of its activity after incubation at 50°C for 30 min (Wang et al., 2014), while *Chlorella sorokiniana* Grx retained at least 80% of maximal activity when incubated at 50°C for 1 h (Chuang et al., 2014). This difference suggests that Ps-Grx3 is a thermostable enzyme, as observed for other cold-adaptive enzymes.

### The Effects of Different Reagents and Chemicals on Ps-Grx3

**FIGURE 7:**
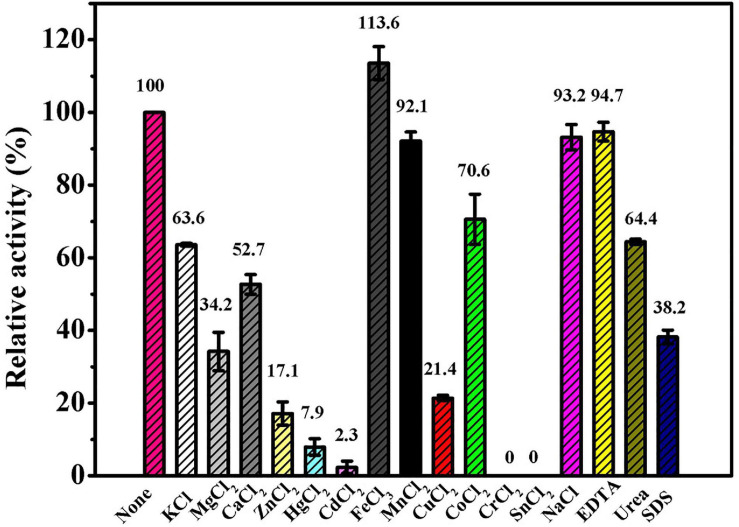
The effects of different reagents on the Ps-Grx3 activity. The above experiments were carried out three times.

The results of the effects of different compounds on Ps-Grx3 activity are shown in Figure 7. In our study, Ps-Grx3 was deactivated by exposure to 5 mM concentrations of Sn^2+^ and Cr^2+^. In the presence of 5 mM Hg^2+^ and Zn^2+^, Ps-Grx3 retained less than 20% activity. Zn^2+^ has also been reported to inhibit the activity of Grx3 from *Pseudoalteromonas* sp. ANT178 (Wang et al., 2014). The addition of other metals, such as Na^2+^ and Mn^2+^, partially inhibited Ps-Grx3 activity, whereas the opposite occurred with the addition of urea and SDS. Notably, exposure to Fe^3+^ also increased the activity of Ps-Grx3.

**FIGURE 8:**
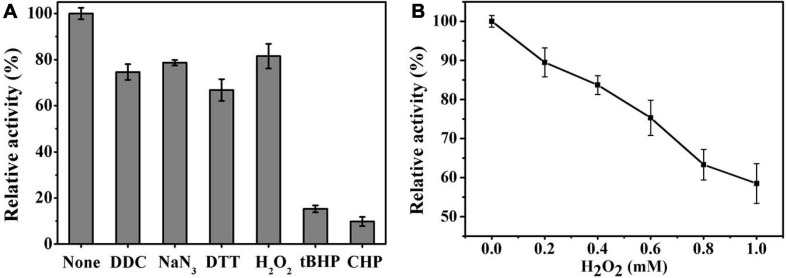
**(A)** The effect of chemicals on Ps-Grx3 activity. Inhibition profiles of Ps-Grx3 after treatment with 0.5 mM DDC, NaN_3_, DTT, H_2_O_2_, tBHP, and CHP. Relative Ps-Grx3 activity was estimated by considering the activity without chemicals-point as 100%. **(B)** Inactivation of Ps-Grx3 by H_2_O_2_. Ps-Grx3 was treated with 0–1.0 mM H_2_O_2_ followed by an analysis of its catalytic activity using the standard HED assay. Enzyme activity was expressed as a percentage of the initial activity. The experiments were carried out three times.

Ps-Grx3 exhibited differential responses to different chemicals (Figure 8A). The activity of Ps-Grx3 was inhibited by NaN_3_ and DTT, but Ps-Grx3 remained 66.8% active after treatment with 0.5 mM DTT. In contrast, Grx from *Chlorella sorokiniana* remained 94.0% active when treated with 1 mM DTT (Chuang et al., 2014). This demonstrated that Ps-Grx3 was more sensitive to DTT than *Chlorella sorokiniana* Grx. DTT is a reducing agent with the capacity to reduce Grx isoforms and proteins are active in their reduced state. Unexpectedly, the activity of Ps-Grx3 was reduced following preincubation with 0.5 mM DTT. This result suggested that DTT might affect either the activity of either GR or GSH (Rendon et al., 2004; Ates et al., 2009). Furthermore, Ps-Grx3 retained 15.3 and 9.8% activity following treatment with tBHP and CHP, respectively, which represented a significant reduction in activity when compared with H_2_O_2_ treatment. This result was in line with that of a previous study (Fernandes and Holmgren, 2004). Of all the chemicals tested, Ps-Grx3 was least sensitive to H_2_O_2._ Ps-Grx3 lost 10 and 25% of its activity in the presence of 0.2 mM and 0.6 mM H_2_O_2_, respectively, but then retained at least 40% activity even when the H_2_O_2_ concentration was raised to 1 mM (Figure 8B).

### The Kinetic Parameters of Ps-Grx3

**FIGURE 9:**
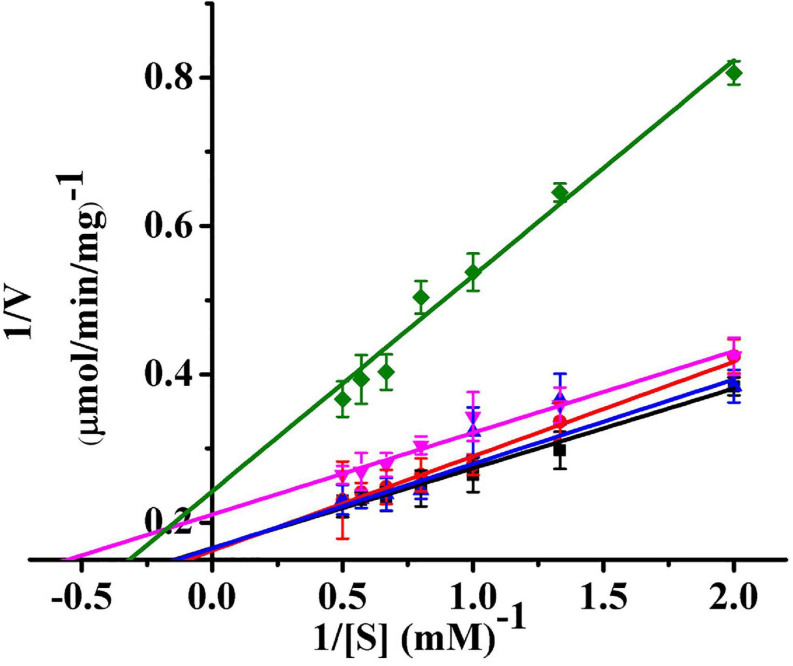
Lineweaver–Burk double reciprocal plots of Ps-Grx3 with respect to DHA (■), S-sulfocysteine (⚫), HED (▲), L-cystine (▼) and BSA (◆). Enzyme activity was measured as described above. All the experiments were performed in triplicate.

The kinetic parameters (*K*_*m*_, *V*_*m*_, and *k*_*cat*_) of Ps-Grx3 are shown in Figure 9 and the kinetic parameter values for the various forms of Ps-Grx3 are summarized in Table 5. The *K*_*m*_ values for L-cystine, HED, and S-sulfocysteine were 0.52, 0.69, and 0.79 mM, respectively. The *K*_*m*_ values for HED and S-sulfocysteine were reported to be 1.68 and 1.77 mM for human Grx2 (Gladyshev et al., 2001). The *K*_*m*_ values of Ps-Grx3 for S-sulfocysteine and BSA were higher, whereas those for L-cystine, HED, and DHA were relatively lower. Ps-Grx3 has a relatively low *K*_*m*_ value for HED (0.69 mM), suggesting that Ps-Grx3 can efficiently reduce the disulfide bond. Additionally, the *k*_*cat*_/*K*_*m*_ values for DHA and BSA were 68.40 and 25.55 1/s/mm However, the highest *V*_*m*_ and *k*_*cat*_ values at the same enzyme concentration were observed with S-sulfocysteine. The *k*_*cat*_ of Ps-Grx3 was different with the five substrates. Ps-Grx3 displayed the highest efficiency with S-sulfocysteine, followed by HED, and then DHA.

**TABLE 5:** The kinetics parameters of the Ps-Grx3.

**Oxidants (0.5 mM)**	***K*_*m*_ (mM)**	***V*_*m*_ (μmol/min/mg)**	***k*_*cat*_ (1/s)**	***k*_*cat*_ /*K*_*m*_ (1/s/mM)**
DHA	0.65	6.03	44.46	68.40
*S*-sulfocysteine	0.79	6.17	45.47	57.56
HED	0.69	6.04	44.57	64.59
L-cystine	0.52	4.74	34.94	67.19
BSA	1.19	4.13	30.41	25.55

Using HED (0.5–2.0 mM) as the substrate, the kinetic parameters of Ps-Grx3 were measured at 25°C, and the results were compared with those of Grxs from different sources (Supplementary Table 1). The results showed that the apparent *V*_*m*_ at 25°C was 6.04 μmol/min/mg for Ps-Grx3 and 188.68 μmol/min/mg for *Taiwanofungus camphorata* Grx. The *k*_*cat*_ represents the maximum number of substrate molecules converted per second for a single catalytic site at a given enzyme concentration. The *k*_*cat*_ and physiological efficiency or specific constant (*k*_*cat*_/*K*_*m*_) of Ps-Grx3 were 44.57 1/s and 64.59 1/s/mM, respectively, under optimum conditions (25°C, pH 8.0). For *Taenia solium* Grx using HED as a substrate, the *k*_*cat*_ and *k*_*cat*_/*K*_*m*_ values were reported to be 5.6 1/s and 7.8 1/s/mM, respectively. A lower *K*_*m*_ is suggestive of a higher affinity toward for a substrate. A *K*_*m*_ value of 0.1 mM has been reported for *E. coli* Grx using HED as substrate, which was lower than that for Ps-Grx3 (0.69 mM), indicating that Ps-Grx3 has a lower affinity for HED when compared with *E. coli* Grx.

### The Ability of Ps-Grx3 to Protect Supercoiled DNA

**FIGURE 10:**
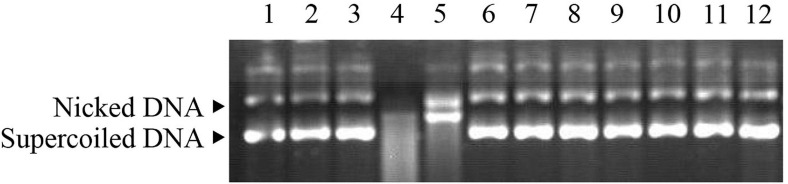
MCO assay demonstrating the antioxidant properties of Ps-Grx3. Lane 1, pUC19 plasmid; lane 2, pUC19 + FeCl_3_; lane 3, pUC19 + DTT; lane 4, pUC19 + FeCl_3_ + DTT; lane 5, pUC19 + FeCl_3_ + DTT + BSA; lane 6, pUC19 + FeCl_3_ + DTT + purified protein (6.25 μg/mL) incubated at 15°C for 1.5 h; lane 7, pUC19 + FeCl_3_ + DTT + purified protein (6.25 μg/mL) incubated at 25°C for 1.5 h; lane 8, pUC19 + FeCl_3_ + DTT + purified protein (6.25 μg/mL) incubated at 25°C for 2 h; lane 9, pUC19 + FeCl_3_ + DTT + purified protein (6.25 μg/mL) incubated at 25°C for 2.5 h; lane 10, pUC19 + FeCl_3_ + DTT + purified protein (12.5 μg/mL) incubated at 25°C for 2.5 h; lane 11, pUC19 + FeCl_3_ + DTT + purified protein (25 μg/mL) incubated at 25°C for 2.5 h; lane 12, pUC19 + FeCl_3_ + DTT + purified protein (50 μg/mL) incubated at 25°C for 2.5 h.

The ability of Ps-Grx3 to protect DNA from nicking was assessed using the MCO DNA cleavage protection assay. This assay involves ROS-mediated DNA disruption, which results in a single-strand break in supercoiled plasmid DNA (Revathy et al., 2015). Here, the pUC19 plasmid was used to investigate the DNA-protective activity of Ps-Grx3 (Figure 10). When the MCO assay components were not all added (lanes 2 and 3), the supercoiled DNA of pUC19 was not damaged, as indicated by the equal ratios of supercoiled: nicked DNA in the control sample (lane 1). However, after the simultaneous addition of DTT and FeCl_3_, the pUC19 DNA showed extensive shredding (lane 4). When BSA was added with the MCO reagents (lane 5), the supercoiled DNA was completely nicked, indicating that BSA had no DNA-protective ability. Conversely, as the concentration of Ps-Grx3 increased (lanes 9–12), the proportion of nicked DNA decreased. Interestingly, at the same, Ps-Grx3 concentration, but at a lower temperature (15°C) and with a shorter incubation time (1.5 h), Ps-Grx3 was also capable of reducing the percentage of nicked DNA (lanes 6 and 9). This indicated that supercoiled pUC19 DNA was sheared by the addition of MCO assay reagents, but that this effect could be reversed by the addition of Ps-Grx3, which reflected its antioxidant activity. The results further implied that the effectiveness of the antioxidant activity of Ps-Grx3 might be closely linked to temperature and duration of incubation, as previously observed for peroxidase (Godahewa et al., 2016). Combined, these results indicated that the newly identified Ps-Grx3 is capable of protecting supercoiled DNA from oxidation-induced damage at low temperatures.

## Conclusion

We successfully cloned a glutaredoxin gene from *Psychrobacter* sp. ANT206 (*Ps-grx3*) and prepared the associated recombinant protein. Characterization of Ps-Grx3 identified it as a cold-adapted enzyme. Cys13, Pro14, and Cys16 were indispensable for its catalytic activity. Among the substrates tested, S-sulfocysteine yielded the highest *V*_*m*_ and *k*_*cat*_ values at the same enzyme concentration. *In vitro*, Ps-Grx3 could protect supercoiled DNA from oxidation-induced damage at low temperatures. Additional studies on the characteristics of Ps-Grx3 are expected to reveal the mechanisms underlying the responses of *Psychrobacter* sp. ANT206 to cold stress.

## Data Availability Statement

The raw data supporting the conclusions of this article will be made available by the authors, without undue reservation.

## Author Contributions

YW, YH, and QW contributed to the conception and design of the study. YW and YH collected the data and performed the experiments. YW and QW wrote the first draft of the manuscript. All authors listed have made a substantial, direct and intellectual contribution to the work, and approved it for publication.

## Conflict of Interest

The authors declare that the research was conducted in the absence of any commercial or financial relationships that could be construed as a potential conflict of interest.

## Footnotes

1 http://www.sbg.bio.ic.ac.uk/phyre2/html/page.cgi?id=index

2 https://saves.mbi.ucla.edu/
