# Supplementary material for: Cloning, Expression, Characterization, and Antioxidant Protection of Glutaredoxin3 From Psychrophilic Bacterium Psychrobacter sp. ANT206
Source: Front Microbiol. 2021 Apr 8;12:633362. doi: 10.3389/fmicb.2021.633362 (PMC8060642; doi:10.3389/fmicb.2021.633362)
Supplement: Supplementary file 1 [file Table_1.docx]

Supplementary Table S1 Comparison kinetic paramaters on Grxs from different sources.

| **Source** | ***Psychrobacter* sp. ANT206 Grx** | ***E.coli* K-12 Grx** | ***Chlorella sorokiniana***  **Grx** | ***Pseudoalter***  ***omonas* sp. ANT178 Grx** | ***Taiwanofungus camphorata* Grx** | ***Taenia solium* Grx** |
| --- | --- | --- | --- | --- | --- | --- |
| *V*m (μmol/min/mg) | 6.04 | - | - | 0.0143 | 188.68 | - |
| *K*_m_ (mM) | 0.69 | 0.1 | 0.17 | 0.46 | 0.57 | 0.72 |
| *k*_cat_ (1/s) | 44.57 | - | 59.44 | - | 27.2 | 5.6 |
| *k*_cat_/*K*_m_ (1/s/mM) | 64.59 | - | 349.7 | - | 47.7 | 7.8 |
| Reference | This study | (Aslund F et al., 1996) | (Chuang et al., 2014) | (Wang et al., 2014) | (Ken et al., 2009) | (Nava et al., 2019) |

Ken, C. F., Lin, C. Y., Jiang, Y. C., Wen, L., and Lin, C. T. (2009). Cloning, expression, and characterization of an enzyme possessing both glutaredoxin and dehydroascorbate reductase activity from *Taiwanofungus camphorata*. J. Agric. Food Chem. 57, 10357–10362. doi: 10.1021/jf9021256
