# Supplementary material for: Cloning, Expression, Characterization, and Antioxidant Protection of Glutaredoxin3 From Psychrophilic Bacterium Psychrobacter sp. ANT206
Source: Front Microbiol. 2021 Apr 8;12:633362. doi: 10.3389/fmicb.2021.633362 (PMC8060642; doi:10.3389/fmicb.2021.633362)
Supplement: Supplementary file 2 [file Image_1.pdf]

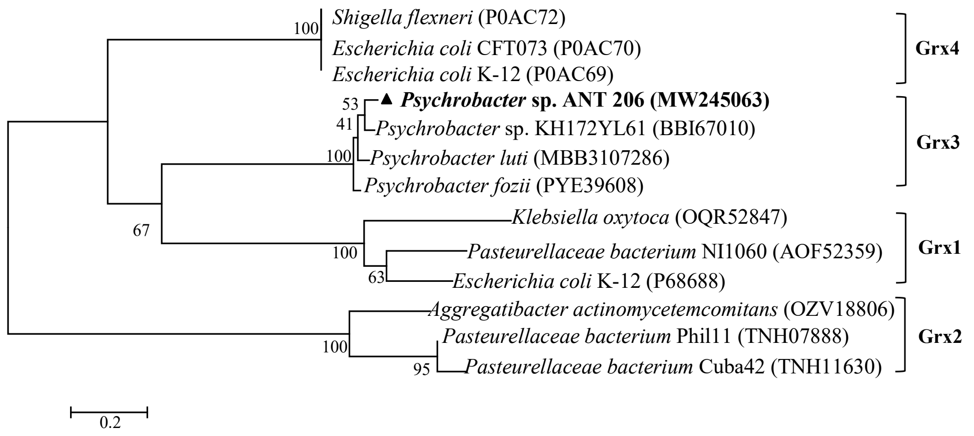

Supplementary Figure S1: Phylogenetic tree of Grx informs was constructed to illustrate the relationship between them using the neighbor-joining method. Node values represent percent bootstrap confidence derived from 1000 replicates. The scale bar is 0.2. The GenBank accession numbers are listed in brackets.
